# Protocols for Randomized Controlled Trials: Advancing Transparency in Infectious Diseases

**DOI:** 10.1093/ofid/ofag163

**Published:** 2026-04-28

**Authors:** Jean-Jacques Parienti, Roger Bedimo, Cynthia L Sears, Paul E Sax

**Affiliations:** Department of Clinical Research, Caen University Hospital, Caen, France; Division of Infectious Diseases, University of Texas Southwestern Medical Center, Dallas, Texas, USA; Department of Oncology, Sidney Kimmel Comprehensive Cancer Center, Johns Hopkins University School of Medicine, Baltimore, Maryland, USA; Division of Infectious Diseases, Brigham and Women's Hospital, Boston, Massachusetts, USA

The first modern clinical trial was conducted in 1948. People with pulmonary tuberculosis were randomly allocated to receive streptomycin or the standard care at that time, which was bed rest. This landmark study not only demonstrated the efficacy of streptomycin but also established the framework for rigorous trial design that continues to guide clinical decisions today. Since then, trials have optimized antiretroviral therapy in people with human immunodeficiency virus, informed strategies to prevent *Clostridioides difficile* infections, and guided treatments during the coronavirus disease of 2019 pandemic. Well-conducted trials improve standards of care and save lives.

Yet even carefully designed trials can be misinterpreted without transparency. Increasingly complex designs with group randomization; multiple, hierarchical, or composite endpoints; and sophisticated modeling approaches make it difficult to distinguish based on the manuscript alone what was planned *a priori* from what emerged after looking at the data. Trial registration is essential but rarely provides sufficient detail to evaluate analytic choices or deviations from the original plan. Without access to the protocol, reviewers and readers are left to infer correct distinction between hypothesis-supporting and hypothesis-generating results. Shifts such as endpoint switching, selective emphasis on favorable secondary outcomes, or post hoc analyses presented without clear labeling can alter interpretation.

At *Clinical Infectious Diseases*, the *Journal of Infectious Diseases*, and *Open Forum Infectious Diseases*, we are committed to ensuring that trials are designed, reviewed, and reported with the highest standards of scientific integrity. Existing reporting frameworks such as CONSORT (CONsolidated Standards Of Reporting Trials) remain invaluable, but they cannot substitute for direct access to the document that defines the study itself: the protocol.

For this reason, the Infectious Diseases Society of America journals will now require that all manuscripts reporting the findings of a randomized controlled trial are submitted with the complete study protocol, including all amendments (or provide a link if previously published). While not mandatory as for randomized controlled trials, authors of Target Trial Emulation studies are strongly encouraged to submit or reference a prespecified study protocol. Protocols will be available online as supplementary material with accepted articles. This step will allow editors and peer reviewers to verify prespecified endpoints and hypotheses, assess sample size assumptions and statistical analysis plans, and clearly distinguish planned analyses from post hoc exploration. It will also encourage balanced reporting of both efficacy and safety outcomes.

This policy is not intended to add administrative burden, since clinical trial protocols exist for funders, ethics committees, or registries. Rather, it reflects a simple principle: Research that guides clinical decisions should be fully transparent. Ready access to protocols benefits reviewers, strengthens reproducibility, supports systematic reviews and meta-analyses, and provides an educational resource for investigators designing future trials.

We believe this requirement will enhance trust in the evidence base and attract studies that share our commitment to rigor and openness. As stewards of the infectious diseases literature, we will continue to refine our processes and encourage other journals to join us in advancing transparent, high-quality clinical research.

Jean-Jacques Parienti, Methodological and Statistical Editor, *Clinical Infectious Diseases*.

Roger Bedimo, Editor-in-Chief, *Open Forum Infectious Diseases*.

Cynthia L. Sears, Editor-in-Chief, *The Journal of Infectious Diseases*.

Paul E. Sax, Editor-in-Chief, *Clinical Infectious Diseases*.

